# Inhibition of lysophosphatidic acid receptor ameliorates Sjögren's syndrome in NOD mice

**DOI:** 10.18632/oncotarget.15916

**Published:** 2017-03-06

**Authors:** Eunhye Park, Donghee Kim, Song Mi Lee, Hee-Sook Jun

**Affiliations:** ^1^ Lee Gil Ya Cancer and Diabetes Institute, Gachon University, Incheon, Republic of Korea; ^2^ College of Pharmacy and Gachon Institute of Pharmaceutical Science, Gachon University, Incheon, Republic of Korea; ^3^ Gil Medical Research Institute, Gil Hospital, Incheon, Republic of Korea

**Keywords:** Sjögren's syndrome, LPA, Ki16425, NOD mice, IL-17

## Abstract

Lysophosphatidic acid (LPA), a bioactive lysophospholipid, is involved in the pathogenesis of chronic inflammatory and autoimmune diseases. In this study, we investigated the role of LPA/LPA receptor (LPAR) signaling in the pathogenesis of Sjögren's syndrome (SS). We found that autotaxin, an LPA producing enzyme, and LPAR1 and LPAR3 mRNA, and IL-17 mRNA were highly expressed in the exocrine glands of 20-week-old nonobese diabetic (NOD) mice, which show SS symptoms at this age, as compared with non-symptomatic 8-week-old NOD mice. In an adoptive transfer model using NOD lymphocytes, treatment with Ki16425, an LPAR1/3 antagonist, restored tear and saliva secretion and decreased symptoms of SS compared with the vehicle-treated group. IL-17 levels in serum and lacrimal glands were also significantly reduced by Ki16425 in recipient mice. In addition, Ki16425 treatment of 20-week-old NOD mice, which spontaneously developed SS, restored saliva volume. Treatment of NOD splenocytes with LPA induced the expression of IL-17 in a dose-dependent manner, and Ki16425 inhibited this increase. LPA stimulated the activation of ROCK2 and p38 MAPK; and inhibition of ROCK2 or p38 MAPK suppressed LPA-induced IL-17 expression. Our data suggest that LPAR signaling stimulates SS development by induction of IL-17 production via ROCK and p38 MAPK pathways. Thus, LPAR inhibition could be a possible therapeutic strategy for SS.

## INTRODUCTION

Sjögren's syndrome (SS) is a chronic autoimmune disease characterized by infiltration of lymphocytes mainly into the salivary glands and lacrimal glands leading to the development of xerostomia (dry mouth) and keratoconjunctivitis sicca (dry eyes) through loss of saliva and tear secretion [1–3].

It was reported that the predominant infiltrating lymphocytes in the salivary glands of patients with SS are CD4^+^ T cells rather than CD8^+^ T cells [4]. Although earlier studies demonstrated the involvement of both Th1 and Th2 cells as triggers of SS onset [5–7], recently Th17 cells and their cytokines such as IL-17 and IL-22 have been recognized as having an important role in the pathogenesis of SS in animal models and humans [8–10]. Also, IL-17 expression is increased in salivary glands and tears of patients with SS compared with healthy controls [11–13].

Lysophosphatidic acid (LPA), a small bioactive lysophospholipid, has been implicated in autoimmune diseases such as rheumatoid arthritis and multiple sclerosis. LPA is mainly produced by the action of an autotaxin, which removes the choline group from lysophosphatidylcholine [14], and mediates cellular responses via a G-protein coupled receptor, LPA receptor 1-6 (LPAR1-6), to trigger specific downstream signaling pathways [15, 16]. LPA/LPAR signaling promotes the production of inflammatory cytokines such as IL-6 and IL-8 in cancer cells [17, 18]. It is known that locally generated LPA facilitates T cell entry into lymph nodes [19], and LPA induces rapid polarization of naïve T cells [19]. Miyabe and colleagues reported that the expression of LPAR1 is increased in fibroblast-like synoviocytes from patients with rheumatoid arthritis, and LPA induces the production of inflammatory cytokines by synoviocytes, suggesting that these cells are activated during rheumatoid arthritis via LPA-LPAR1 signaling [20, 21]. Also, blockade of LPAR signaling by an antagonist of LPAR1/3, reduces symptoms of rheumatoid arthritis in an animal model [22]. In addition, autotaxin activity and LPA levels are significantly higher in patients with multiple sclerosis compared with healthy controls [23, 24]. In an animal model of multiple sclerosis, LPAR1 deletion effectively protects against dorsal root injury [25]. This evidence emphasizes that LPAR signaling plays an important role in promoting inflammation-associated autoimmune disease. However, it is not known yet whether LPAR signaling is involved in the development of SS.

In this study, we investigated the effects of inhibition of LPAR signaling on the development of SS in nonobese diabetic (NOD) mice using Ki16425, an LPAR1/3 antagonist.

## RESULTS

### Expression of autotaxin, LPARs, and inflammatory cytokines was increased in exocrine glands of 20-week-old NOD mice

**Figure 1:**
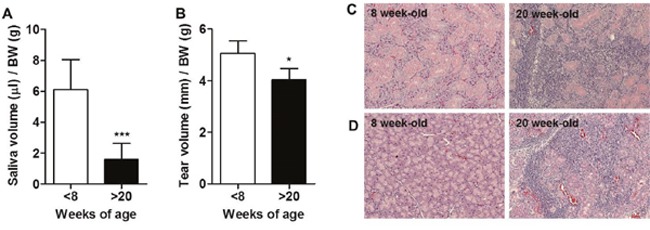
Development of Sjögren's syndrome (SS) occurred in over 20-week-old male NOD mice **(A)** Saliva and **(B)** tear secretion was stimulated by injection of 1 or 5 mg/kg pilocarpine, respectively, and measured in 7 to 8- and 20 to 25-week-old male NOD mice. Saliva and tear secretion was normalized by body weight (BW) for each mouse (n=9-10/group). Means ± SD (*p<0.05, ***p<0.001). **(C)** Salivary glands and **(D)** lacrimal glands were harvested from 8- or 20-week-old male NOD mice. Paraffin sections were prepared and stained with hematoxylin and eosin.

Loss of secretory function by destruction of exocrine glands in NOD mice occurs at about 20 weeks of age [3]. Thus, we first confirmed that saliva (Figure 1A) and tear (Figure 1B) secretion was significantly decreased, and lymphocytic infiltration in the salivary gland (Figure 1C) and lacrimal gland (Figure 1D) was increased in 20-week-old NOD mice compared to 8-week-old mice.

**Figure 2:**
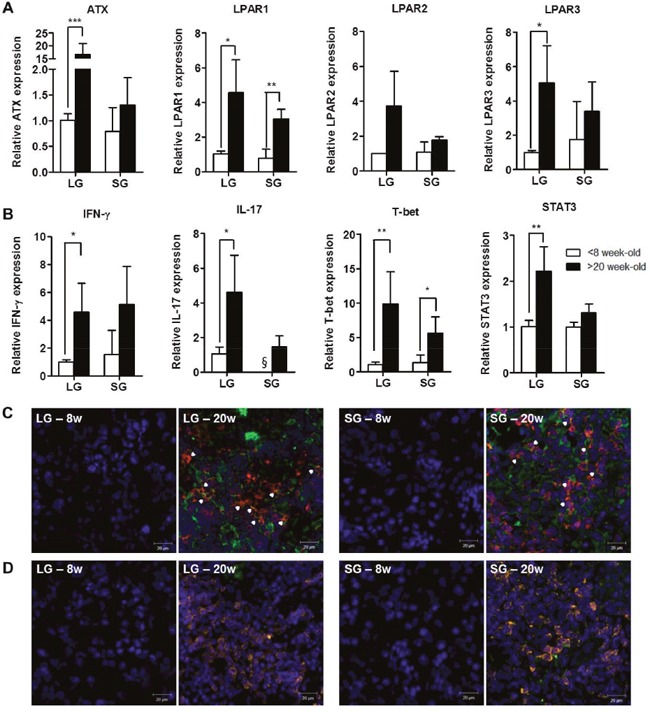
Expression of autotaxin, LPARs, and IL-17 was increased in exocrine glands from NOD mice with SS Total RNA was isolated from lacrimal glands (LG) and salivary glands (SG) of 8- or 20-week-old NOD male mice. The mRNA expression of **(A)** autotaxin (ATX), LPAR1, LPAR2, and LPAR3 and **(B)** IFN-γ, IL-17, T-bet and STAT3 was detected by qRT-PCR (n=3-7/group). Means ± SD (*p<0.05, **p<0.01, ***p<0.005, §, not detected). **(C-D)** Representative pictures of immunofluorescence staining for LPAR1 **(C)** and IL-17 **(D)** expression. Lacrimal and salivary glands of NOD mice were stained for CD3^+^ T (red), LPAR1 (green) or IL-17 (green) antibodies with DAPI nuclei staining (blue) at 8 or 20 weeks of age. Arrowheads and yellow color indicate double-positive cells. Scale bars: 20 μm.

To investigate any correlation between disease progression and LPAR expression, we analyzed the expression of autotaxin and LPAR1-5 in lacrimal and salivary glands of 8- and 20-week-old NOD mice. We found that autotaxin mRNA was significantly increased in lacrimal glands from 20-week-old NOD mice compared with 8-week-old mice (Figure 2A). The mRNA expression of LPAR1 and LPAR3 was significantly elevated in lacrimal glands of 20-week-old mice compared with 8-week-old mice. LPAR1 was also significantly elevated in the salivary glands of older mice, but not LPAR2 or LPAR3 (Figure 2A). Neither LPAR4 nor LPAR5 mRNA expression was detected (data not shown). IFN-γ and IL-17 mRNA expression was significantly increased in lacrimal glands of 20-week-old NOD mice compared with 8-week-old NOD mice (Figure 2B). mRNA levels of T-bet and signal transducer and activator of transcription (STAT) 3, specific transcription factors for Th1 and Th17 cells, respectively, were also significantly increased in lacrimal glands from older mice (Figure 2B). The expression of IL-17 and LPAR1 in lacrimal (p = 0.01) and salivary (p = 0.016) glands was positively correlated. Immunohistochemical analysis of exocrine glands revealed that CD3^+^ T cells were highly infiltrated and some T cells expressed LPAR1 in NOD mice at 20 weeks of age, but not at 8 weeks (Figure 2C). LPAR1 expression was also observed in CD3-negative cells (Figure 2C). In addition, IL-17 was mainly expressed in infiltrated CD3^+^ T cells from gland tissues in NOD mice at 20 weeks of age (Figure 2D).

### LPAR antagonist treatment attenuated symptoms of SS in an adoptive transfer model

**Figure 3:**
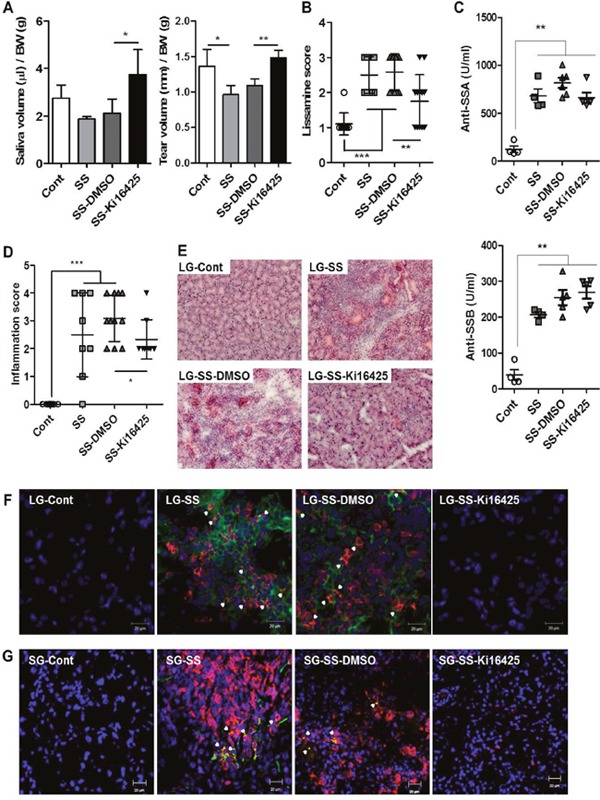
Ki16425 treatment attenuated symptoms of SS in an adoptive transfer model Splenocytes and superficial cervical lymph node cells were transferred intoNOD scid mice, and mice were injected daily with 15 mg/kg Ki16425 for 4 weeks. **(A)** Eight weeks after adoptive transfer, pilocarpine-stimulated saliva and tear secretion was measured and normalized by body weight. **(B)** Eight weeks after adoptive transfer, the eyes were stained with lissamine green B and ocular surface damage was scored. **(C)** Autoantibodies for anti-SSA and anti-SSB were detected by ELISA in serum from recipient NOD scid mice. **(D)** Lacrimal glands were isolated from recipient mice, stained with hematoxylin and eosin and scored for focal inflammation. Each dot represents an individual mouse. Values and bars are means ± SD (*p<0.05, **p<0.01, ***p<0.005). Cont, neither adoptive transferred nor injected (n=5); SS, only adoptive transferred (n=4-7); SS-DMSO, adoptive transferred and injected with vehicle (n=6-11); SS-Ki16425, adoptive transferred and injected with Ki16425 (n=6-7). **(E)** Representative pictures of hematoxylin and eosin staining in lacrimal glands. **(F-G)** Lacrimal glands **(F)** and salivary glands **(G)** from recipient NOD scid mice were stained using CD3^+^ T (red) and LPAR1 (green) antibodies with DAPI nuclei staining (blue). Arrowheads indicate double-positive cells. Scale bars: 20 μm.

To investigate whether an LPAR antagonist can ameliorate the development of SS, the adoptive transfer model was used as previously described [26, 27]. We transferred a mixture of splenocytes and superficial cervical lymph node cells from 20 ∼ 25-week-old NOD mice into NOD scid mice. Recipient NOD scid mice were injected daily with 15 mg/kg Ki16425, an antagonist for LPAR1/3, for 4 weeks. Four weeks after the last injection, saliva and tear secretion was measured. Saliva and tear secretion was reduced in SS induced recipients (SS) and vehicle-treated recipients (SS-DMSO) compared with control NOD scid mice that did not receive NOD lymphocytes (Figure 3A). However, saliva and tear secretion was restored by Ki16425 treatment (SS-Ki16425) compared with SS-DMSO (Figure 3A). Also, loss of tear secretion leads to epithelial damage of the ocular surface [28]. Thus, we measured damage to the ocular surface by lissamine green B staining. While the lissamine score was increased in the SS-DMSO group compared with the control group, Ki16425 treatment significantly decreased this score compared with the SS-DMSO group (Figure 3B). Meanwhile, anti-SSA and anti-SSB autoantibody levels in sera were significantly increased in the SS and SS-DMSO groups, but Ki16425 treatment did not change the levels of these autoantibodies (Figure 3C). Consistent with the functional restoration of the lacrimal gland, the inflammation score in the lacrimal glands was decreased in the SS-Ki16425 group compared with the SS-DMSO group (Figure 3D). Lymphocytic infiltration in the lacrimal glands was also decreased in the SS-Ki16425 group compared with the SS-DMSO group (Figure 3E). Immunohistochemical analysis of LPAR1 expression in lacrimal glands (Figure 3F) and salivary glands (Figure 3G) showed that CD3/LPAR1 double-positive cells were increased in the SS and SS-DMSO groups compared with the control group. Ki16425 treatment decreased these double-positive cells compared to the SS-DMSO group (Figure 3F and 3G).

### LPA receptor antagonist treatment reduced serum IL-17 levels and IL-17 expression in exocrine glands in an adoptive transfer model

**Figure 4:**
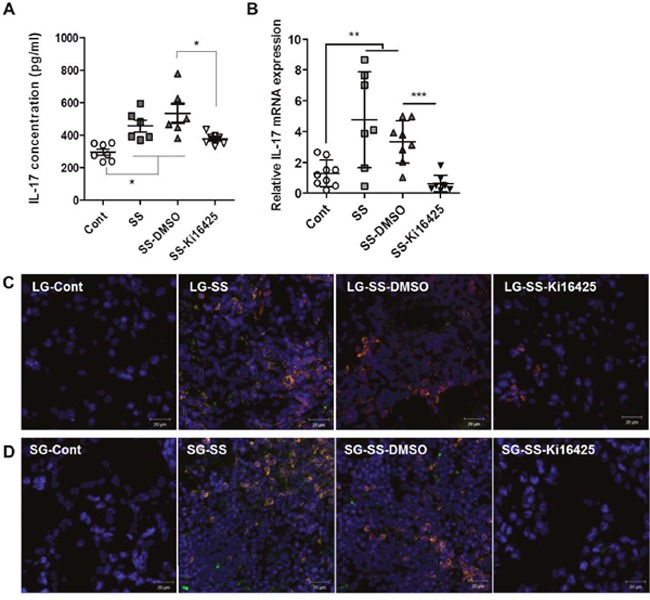
Ki16425 treatment reduced IL-17 expression in an adoptive transfer SS model Splenocytes and superficial cervical lymph node cells were transferred intoNOD scid mice and mice were either not injected (SS) or injected with DMSO (SS-DMSO) or Ki16425 (SS-Ki16425). **(A)** Eight weeks after adoptive transfer, serum IL-17 content in recipient mice was detected by ELISA. **(B)** IL-17 mRNA expression was analyzed in the lacrimal glands of recipient mice by qRT-PCR. Values are means ± SD (n= 6-9/group, *p<0.05, **p<0.01, ***p<0.005). **(C-D**) Lacrimal glands (C) and salivary glands (D) from recipient NOD scid mice were stained for CD3^+^ T (red) and IL-17 (green) antibodies with DAPI nuclei staining (blue). Yellow color indicates double-positive cells. Scale bars: 20 μm.

To investigate the effects of Ki16425 on IL-17 expression *in vivo*, we analyzed serum IL-17 levels and IL-17 mRNA expression in the lacrimal glands from adoptively transferred NOD scid mice. Serum IL-17 (Figure 4A) and IL-17 mRNA (Figure 4B) levels were significantly increased in the SS and SS-DMSO groups compared with the control group, however Ki16425 treatment (SS-Ki16425) significantly decreased these levels as compared with the SS-DMSO group (Figure 4A and 4B). Immunohistochemical analysis of IL-17 expression in the lacrimal glands (Figure 4C) and salivary glands (Figure 4D) showed that IL-17-expressing CD3^+^ T cells were increased in the SS and SS-DMSO groups compared with the control group. Ki16425 treatment inhibited the increase of these cells compared with the SS-DMSO group (Figure 4C and 4D). These results suggest that LPA/LPAR inhibition decreased T cell infiltration and IL-17 expression in exocrine glands.

### LPA receptor antagonist treatment improved disease symptom in the spontaneous SS NOD model

**Figure 5:**
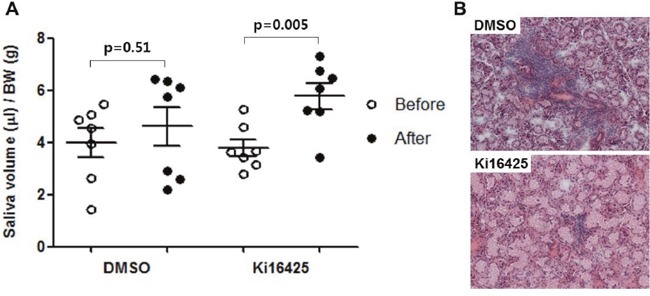
Ki16425 treatment improved disease symptom in the spontaneous SS NOD model Twenty-week-old male NOD mice were injected daily with 15 mg/kg Ki16425 or vehicle (DMSO) for 4 weeks. **(A)** Before and four weeks after injection, pilocarpine-stimulated saliva secretion was measured and normalized by body weight. **(B)** Four weeks after injection, salivary glands were isolated and stained with hematoxylin and eosin. Each dot represents an individual mouse. Values are means ± SD.

To investigate the therapeutic effects of Ki16425 on the spontaneously developed SS, 20-week-old male NOD mice were injected daily with 15 mg/kg Ki16425 or vehicle (DMSO) for 4 weeks and salivary secretion was measured. Ki16425 treatment significantly increased salivary secretion over pre-treatment levels, while salivary secretion in DMSO-treated mice was not changed (Figure 5A). Immune cell infiltration in salivary glands was decreased in the Ki16425 group compared with the DMSO group (Figure 5B).

### LPA treatment induced expression of IL-17 in splenocytes from NOD mice

**Figure 6:**
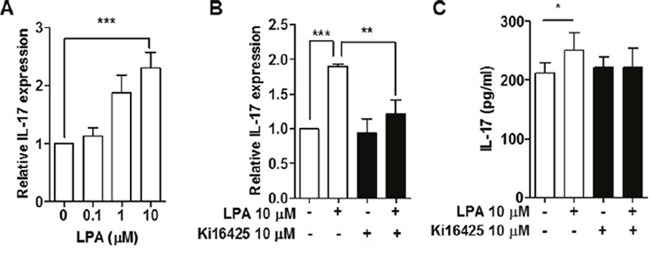
LPA induced expression of IL-17 by LPAR signaling in splenocytes from NOD mice **(A)** Splenocytes were treated with the indicated doses of LPA for 24 h, and mRNA expression of IL-17 was analyzed by qRT-PCR. **(B)** Splenocytes were treated with or without 10 μM LPA and 10 μM Ki16425 for 24 h, and mRNA expression of IL-17 was analyzed by qRT-PCR. **(C)** Under conditions as for (B), splenocytes were re-stimulated with 1 μg/ml PMA and 2 μg/ml ionomycin for 6 h, and the supernatants were analyzed for IL-17 by ELISA. Means ± SD (n=3/group, *p<0.05, **p<0.01, ***p<0.001).

To investigate the effect of LPA on the expression of IL-17 mRNA in lymphocytes, we measured mRNA expression of IL-17 in splenocytes treated with LPA. IL-17 mRNA expression was increased by LPA in a dose-dependent manner (Figure 6A). To examine whether the increased IL-17 mRNA expression is truly mediated by LPAR signaling, we measured IL-17 mRNA expression after treatment with Ki16425 and found that Ki16425 inhibited LPA-induced IL-17 mRNA expression (Figure 6B). Consistent with this result, Ki16425 treatment also inhibited LPA-induced IL-17 secretion (Figure 6C). These results suggest that LPA induces IL-17 expression through LPAR signaling.

### LPA-induced IL-17 expression was mediated by ROCK2 and p38 MAPK activation

**Figure 7:**
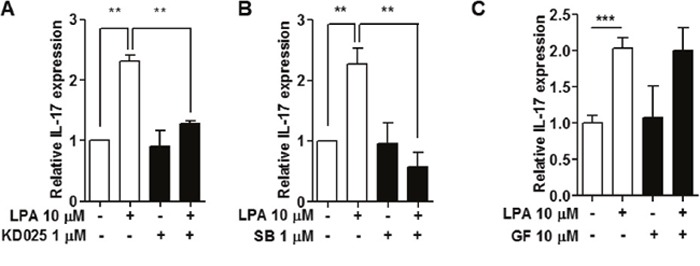
Inhibition of ROCK2 and p38 MAPK suppressed LPA-induced IL-17 expression Splenocytes from NOD mice were pre-treated with **(A)** a ROCK2 inhibitor, 1 μM KD025 for 1 h, **(B)** a p38MAPK inhibitor, 1 μM SB203580 (SB) for 30 min, or **(C)** a PKC inhibitor, 10 μM GF109203X for 30 min, and then 10 μM of LPA was added. IL-17 mRNA expression was analyzed by qRT-PCR. Means ± SD (n=3/group, **p<0.01, ***p<0.001).

LPAR signaling activates ROCK, PKC or p38 MAPK pathways [29]. To investigate which specific signaling pathways are involved in LPA-stimulated IL-17 expression in NOD splenocytes, we first used specific inhibitors, KD025 for ROCK2, SB203580 for p38 MAPK, and GF109203X for PKC. KD025 (Figure 7A) and SB203580 (Figure 7B) treatment suppressed LPA induced IL-17 expression, but GF109203X treatment did not (Figure 7C).

**Figure 8:**
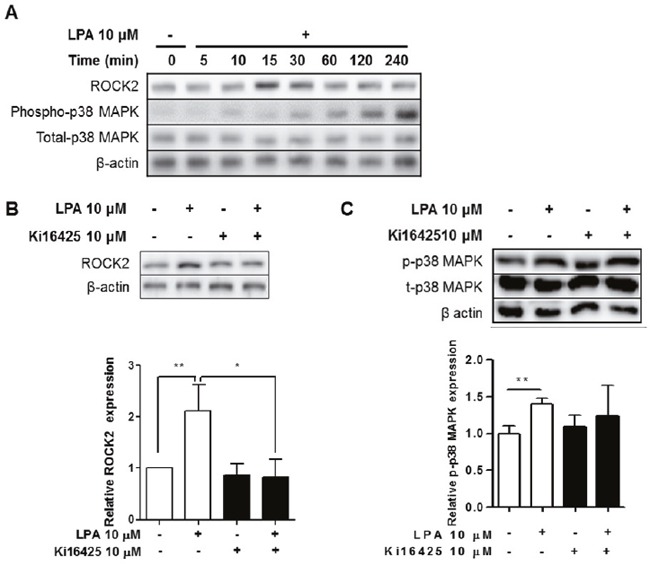
LPA induced ROCK2 and p38 MAPK activation via LPAR signaling in splenocytes **(A)** Splenocytes were treated with 10 μM LPA for the indicated times. ROCK2, phospho-p38 MAPK, total-p38 MAPK, and β-actin were analyzed by western blot. **(B and C)** Splenocytes were treated with or without 10 μM LPA and 10 μM Ki16425 for 15 min or 120 min. ROCK2 (B), p38 MAPK (C) and β-actin were analyzed by western blot (upper panel) and quantified by ImageJ Software (lower panel). Means ± SD (n=3/group, *p<0.05, **p<0.01).

We measured the expression levels of ROCK2 and phospho-p38 MAPK after LPA treatment. ROCK2 was increased by LPA treatment in a time-dependent manner, with a peak induction at 15 min (Figure 8A). Also, LPA induced phosphorylation of p38 MAPK at later time points - 120 and 240 min after the treatment (Figure 8A). Ki16425 treatment suppressed LPA-induced ROCK2 (Figure 8B) and p38 MAPK expression (Figure 8C). These results indicate that LPA promoted expression of IL-17 via ROCK2/p38 MAPK pathway.

## DISCUSSION

SS is a complicated autoimmune disease with no cure, and the treatment of SS is merely to relieve symptoms. LPAR signaling plays an important role in other inflammation-associated autoimmune diseases. In patients with rheumatoid arthritis, the expression of autotaxin and LPAR1 is increased in fibroblast-like synoviocytes compared with patients with osteoarthritis [20]. Inhibition of LPAR signaling by LA-01, an LPAR1-specific inhibitor, alleviates collagen-induced rheumatoid arthritis and suppresses Th17 differentiation [20]. As well, LPA is elevated in serum of patients with systemic sclerosis [30] or multiple sclerosis [23], suggesting that LPA and LPAR signaling plays an important role in the pathogenesis of autoimmune disease. For this reason, we investigated the possible involvement of LPAR signaling in the development of SS in NOD mice.

We found that autotaxin, LPAR1 and LPAR3 mRNA expression was highly expressed in the lacrimal and salivary glands in 20-week-old NOD mice, a model for spontaneous SS. LPAR1 expression was observed in both CD3^+^ T cells and non-T cells. LPAR1 expression is detected in fibroblast-like synoviocytes [20] and lipopolysaccharide-stimulated alveolar epithelial cells [31], therefore epithelial cells in exocrine tissues in inflammatory environment may also express LPAR1.

The onset age of SS in NOD mice is around 20 weeks, but there are individual differences [3]. In addition, it takes a long experimental period to test the efficacy of the drug. Thus we used the adoptive transfer model to synchronize the onset of the disease. Adoptive transfer of a mixture of superficial cervical lymph node cells and splenocytes from NOD mice into NOD scid mice induced SS, evidenced by functional loss of the exocrine glands, infiltration of immunocytes and the increase of IL-17 expression in exocrine glands. Treatment with Ki16425, a selective LPAR1/3 antagonist, ameliorated SS in this adoptive transfer model. In addition, Ki16425 treatment ameliorated symptoms of spontaneously developed SS in NOD mice. Unexpectedly, Ki16425 treatment in the adoptive transfer model did not change anti-SSA and anti-SSB autoantibody levels at 8 weeks after the treatment with Ki16425. Ki16425 treatment may not affect autoantibody production or antibody changes might not be detectable at this time. It was reported that saliva flow is improved 10 weeks after treatment with an immunosuppressive drug in NOD mice, however, the anti-SSA and anti-SSB antibodies are not decreased until 24 and 16 weeks, respectively [32]. Further studies are required to investigate changes in B cells, plasma cells or follicular helper T cells.

We observed an increased expression of IFN-γ and IL-17 in lacrimal glands with severe infiltration, and blockade of LPAR by Ki16425 suppressed LPA-induced IL-17 expression. LPA/LPAR signaling has been reported to promote the production of inflammatory cytokines such as IL-1β, IL-6 [33], IL-8 [34] and TNF-α [35]. Our results showed that LPA treatment of splenocytes induced IL-17 expression, but not IFN-γ (data not shown). This result is different from a previous report that showed the reduced expression of IFN-γ by Ki16425 treatment in dermal fibrosis in a mouse model of systemic sclerosis [36].

The LPA/LPAR1/3 response is regulated via various intracellular signaling pathways including protein kinases such as ROCK, PKC, and p38 MAPK [37–39]. In addition, these molecular pathways are involved in IL-17 production [40–44]. p38 MAPK signaling regulates IL-17 production in CD4^+^ T cells both in mouse [43] and human [40], and inhibition of p38 MAPK in T cells prevents the development of autoimmune encephalomyelitis [43]. In addition, signaling by PKC-theta stimulates STAT3 expression, contributing to Th17 differentiation in mouse T cells [42], and pharmacological inhibition of PKC in human T cells prevents IL-17 production [41]. As well, ROCK2, but not ROCK1, is known to be involved in IL-17 expression via STAT3 phosphorylation in response to T cell receptor stimulation [44]. Thus, we checked whether these signaling pathways are involved in the induction of IL-17 mRNA expression by LPA. We observed that LPA stimulated the activation of ROCK2, PKC and p38 MAPK, and treatment with Ki16425 inhibited this activation. Specific inhibition of ROCK2 or p38 MAPK blocked LPA-induced IL-17 mRNA expression, but inhibition of PKC did not affect LPA-induced IL-17 mRNA expression, indicating that LPA-induced IL-17 expression may be mediated by ROCK2 and p38 MAPK signaling pathways in our mouse model of SS.

In animal models of rheumatoid arthritis, Miyabe and colleagues showed that LPAR1-mediated signaling is crucial for disease development [20]. Our data revealed that both LPAR1 and LPAR3 mRNA are highly expressed in lacrimal glands and salivary glands of the SS-disease mouse. As Ki16425 is an antagonist of both LPAR1 and LPAR3, we do not know which LPAR, LPAR1 or LPAR3, is involved in the development of SS. In previous reports, LPA induced the Rho/ROCK pathway by LPAR1 or LPAR2 and G_12/13_ protein interaction, but not by LPAR3 [45]. We presume that LPAR1 might play a major role for the development of SS, similar to the rheumatoid arthritis animal model. However, this remains to be tested in a conditional model, such as LPAR1^−/−^ or LPAR3^−/−^ mice.

Based on these results, we conclude that LPAR signaling stimulates the development of SS via IL-17 production. Thus, LPAR inhibition could be a possible therapeutic strategy for SS.

## MATERIALS AND METHODS

### Mice

Male NOD and immunodeficient NOD scid mice were purchased from The Jackson Laboratory (Bar Harbor, ME, USA) and Korea Research Institute of Bioscience and Biotechnology (KRIBB, Daejeon, South Korea), respectively. As male NOD mice show more severe infiltration in the lacrimal glands than females, male NOD mice were used throughout the study. To investigate differences by age, we compared NOD mice 8 weeks of age or younger and 20 weeks of age or older. All mice were maintained under specific pathogen-free conditions at the animal facility of Lee Gil Ya Cancer and Diabetes Institute, Gachon University (Incheon, Korea), and all animal experiments were approved by the Institutional Animal Care and Use Committee at Lee Gil Ya Cancer and Diabetes Institute, Gachon University.

### Measurement of saliva and tear volume

Pilocarpine-stimulated saliva and tear volumes were measured as described previously [46, 47]. Briefly, NOD or NOD scid mice were fasted for 5–7 h and then anesthetized with an intraperitoneal (i.p.) injection of ketamine (25 mg/kg body weight; Huons Co., Kyounggi, Korea). Following anesthesia, NOD mice were injected i.p. with pilocarpine hydrochloride (Sigma-Aldrich, St. Louis, MO, USA) at 1 (0.5 for NOD scid) and 5 (4.5 for NOD scid) mg/kg body weight to induce saliva and tear secretion, respectively. Ten minutes after injection, tear volumes were determined using a phenol red thread (Zone-Quick, Menicon, Tokyo, Japan). Once salivation became visible, fluid from the oral cavity was collected by cotton swabs for 10 minutes. The volume was measured and normalized according to body weight.

### Histological analysis of lacrimal and salivary glands

Lacrimal and salivary glands were removed from NOD or recipient NOD scid mice, fixed in 10% neutral-buffered formalin and embedded in paraffin blocks. Tissue sections (4 μm) were stained with hematoxylin and eosin, and images were analyzed with a light microscope. The histological score was determined as the number of infiltrated foci as follows:score 0, no foci; score 1, less than 1 focus; score 2, 2-5 foci; score 3, 6-9 foci; score 4, over 10 foci. A focus was defined as an infiltrated region (0.04 mm^2^) seen within the histological section. For immunohistochemical analysis, cryosections (5 μm) of lacrimal and salivary gland tissue were stained with PE anti-CD3 (BD Pharmingen, San Diego, CA, USA), anti- LPAR1 (Santa Cruz Biotechnology, Dallas, TX, USA) or anti-IL-17a (Biolegend, San Diego CA, USA) antibodies and 4′-6-diamidino-2-phenylindole (DAPI, Life Technologies, Carlsbad, CA, USA). Images were captured using a laser scanning confocal microscope (Carl Zeiss, Munich, Germany).

### Quantitative real-time PCR (qRT-PCR)

Total RNA extraction and cDNA synthesis was performed as described previously [46]. qRT-PCR analysis was performed by using SYBR Master Mix (TaKaRa, Otsu, Japan) and the CFX384^TM^ Real-Time PCR System (BIO-RAD, Hercules, CA, USA). The relative copy number was calculated using the threshold crossing point (Ct) as calculated by the ΔΔCt calculations. All primer sequences are listed in Supplementary Table 1.

### Isolation of lymphocytes and adoptive transfer

To synchronize the onset of the SS in NOD scid mice, we adoptively transferred splenocytes and superficial cervical lymph node cells using with slight modifications of previously described methods [26, 27]. Twenty to twenty-five-week-old NOD male mice were used as donors. Briefly, splenocytes and superficial cervical lymph node cells were mixed (1 × 10^7^ cells/200 μl) at a ratio of 1:1 and transferred into the tail vein of 6∼8-week-old NOD scid mice. After adoptive transfer, mice were i.p. injected with Ki16425 (15 mg/kg body weight; Biobyt Ltd., Cambridge, UK) or an equivalent volume of DMSO daily for 4 weeks. Groups of recipient mice were classified as follows: Control, neither adoptive transferred nor injected; SS, only adoptive transferred; SS-DMSO, adoptive transferred and injected with vehicle; and SS-Ki16425, adoptive transferred and injected with Ki16425. Eight weeks after adoptive transfer, ocular surface damage and tear and saliva production were measured. Thereafter, the mice were sacrificed.

### Analysis of ocular surface damage

To evaluate the degree of ocular surface damage, one drop of 3% lissamine green B (Sigma) was administered to the inferolateral conjunctival sac of recipient NOD scid mice. The corneal surface was observed with a handheld slit lamp biomicroscope (Kowa, Nagoya, Japan). Dye staining of the cornea was scored as follows: 0 for no stained spots; 1 for some stained spots; 2 for many separate stained spots; and 3 for many stained spots appearing close together [48].

### Determination of autoantibodies in serum

Serum was collected from recipient NOD scid mice 8 weeks after adoptive transfer. The concentration of autoantibodies against SS-related antigen A (SSA/Ro) and SS-related antigen B (SSB/La) was measured by enzyme linked immunosorbent assay (ELISA) kit (Alpha Diagnostic International Inc., San Antonio, TX, USA) in accordance with the manufacturer's protocol.

### Ki16425 treatment of spontaneous SS NOD mice

Salivary secretion was measured in 20-week-old male NOD mice, and then mice were injected daily with 15 mg/kg Ki16425 or vehicle (DMSO) for 4 weeks. Four weeks after the first injection, salivary production was measured again.

### *In vitro* treatment of cells with LPA and/or Ki16425

Spleens were isolated from male NOD mice and splenocytes were prepared as described previously [46]. Splenocytes (4 × 10^6^) were pre-incubated in 6-well plates in serum-free RPMI 1640 (SFR, Welgene, Daegu, Korea) medium with 5% bovine serum albumin (BSA, Sigma) at 37°C, 5% CO_2_ for 1 h. Splenocytes were stimulated with LPA (0, 0.1, 1 or 10 μM; Avanti Polar Lipids Inc, Alabama, USA) for 24 h. For inhibition of LPAR signaling, splenocytes were stimulated with LPA (10 μM) in the presence or absence of Ki16425 (10 μM) for 24 h. For expression of rho-associated protein kinase (ROCK) 2 and p38 mitogen-activated protein kinase (MAPK), cells were stimulated with LPA (10 μM) for various times. For inhibition of ROCK2, cells were pre-incubated with KD025 (1 μM; Sigma) for 1 h, washed, and then stimulated with LPA (10 μM). For inhibition of p38 MAPK, cells were pre-incubated with SB203580 (1 μM; Enzo Life Sciences, Farmingdale, NY, USA) for 30 min, and then stimulated with LPA (10 μM). For inhibition of PKC, cells were pre-incubated with GF109203X (10 μM) for 30 min and then stimulated with LPA (10 μM). After 24 h of incubation, cells were harvested and assayed.

### Determination of IL-17 levels

To measure plasma IL-17 concentration, serum was collected from recipient NOD scid mice 8 weeks after adoptive transfer. To measure splenic IL-17 secretion, splenocytes (2 × 10^6^) from male NOD mice were seeded in 12-well plates in 5% BSA SFR medium and stimulated with LPA (10 μM) in the presence or absence of Ki16425 (10 μM). After 24 h, the cells were washed and re-stimulated with 1 μg/ml phorbol 12-myristate 13-acetate (PMA, Sigma) and 2 μg/ml ionomycin (Molecular Probes, Invitrogen, Carlsbad, CA, USA) in 0.2% BSA in SFR medium. After 6 h, the culture supernatant was harvested and frozen at −80°C until assayed. IL-17 concentration in serum and cell culture supernatants was measured by LEGEND MAX™ mouse IL-17a ELISA Kit (BioLegend) in accordance with the manufacturer's protocol.

### Western blot analysis

Protein was isolated from LPA-stimulated splenocytes from NOD mice using Mammalian Protein Extraction Buffer (GE Healthcare, Piscataway, NJ, USA) supplemented with protease inhibitors. Proteins were resolved by SDS-PAGE, and western blotting was performed with antibodies against phospho-p38 MAPK (Abcam, Cambridge, UK), total-p38 MAPK (Abcam), ROCK2 and β-actin (Santa Cruz). β-actin was used as a loading control. Signals were detected using Fujifilm luminescent image analyzer LAS4000 with an ECL detection kit. Three or four separate experiments were performed with different samples.

### Statistical analysis

All data were expressed as mean ± SD. Statistical difference was estimated either by Student's t-test or ANOVA followed by post-hoc test. Correlation analysis was performed using the Pearson's correlation matrix on Graphpad Prism 5.01 software. The value of statistical significance was set at *p < 0.05*.

## SUPPLEMENTARY MATERIALS AND TABLE


