# Supplementary material for: Inhibition of lysophosphatidic acid receptor ameliorates Sjögren's syndrome in NOD mice
Source: Oncotarget. 2017 Mar 6;8(16):27240–51. doi: 10.18632/oncotarget.15916 (PMC5432331; doi:10.18632/oncotarget.15916)
Supplement: Supplementary file 1 [file oncotarget-08-27240-s001.pdf]

Inhibition of lysophosphatidic acid receptor ameliorates Sjögren's syndrome in NOD mice

SUPPLEMENTARY MATERIALS

SUPPLEMENTARY TABLE

Supplementary Table 1: Primer sequences for qRT-PCR

Gene symbol	Sequences (5' → 3')	
Cyclophilin B	sense	TGGAGAGCACCAAGACAGACA
	anti-sense	TGCCGGAGTCGACAATGAT
ATX	sense	GACCCTAAAGCCATTATTGCTAA
	anti-sense	GGGAAGGTGCTGTTTCATGT
IFN-γ	sense	CGGCACAGTCATTGAAAGCCTA
	anti-sense	GTTGCTGATGGCCTGATTGTC
IL-17	sense	GTGTCAATGCGGAGGGAA
	anti-sense	TTCAGGACCAGGATCTCTTGCT
T-bet	sense	AATCGACAACAACCCCTTTG
	anti-sense	CGAGGGGACACTCGTATCAA
STAT3	sense	TCCAAGCAGTGATTTCTCCCTAGAAC
	anti-sense	GAGAGGTACTTCTGGTACCTTTTTCC
LPAR1	sense	TCTTCTGGGCCATTTTCAAC
	anti-sense	TGCCTGAAGGTGGCGCTCAT
LPAR2	sense	TCAGCCTAGTCAAGACGGTTG
	anti-sense	CATCTCGGCAGGAATATACCAC
LPAR3	sense	ACACCAGTGGCTCCATCAG
	anti-sense	GTTCATGACGGAGTTGAGCAG
